# Shear Stress-Normal Stress (Pressure) Ratio Decides Forming Callus in Patients with Diabetic Neuropathy

**DOI:** 10.1155/2016/3157123

**Published:** 2016-12-05

**Authors:** Ayumi Amemiya, Hiroshi Noguchi, Makoto Oe, Kimie Takehara, Yumiko Ohashi, Ryo Suzuki, Toshimasa Yamauchi, Takashi Kadowaki, Hiromi Sanada, Taketoshi Mori

**Affiliations:** ^1^Department of Wound Care Management, Graduate School of Medicine, The University of Tokyo, 7-3-1 Hongo, Bunkyo-ku, Tokyo, Japan; ^2^Department of Nursing Physiology, Graduate School of Nursing, Chiba University, 1-8-1 Inohana, Chuo-ku, Chiba-shi, Chiba, Japan; ^3^Department of Life Support Technology (Molten), Graduate School of Medicine, The University of Tokyo, 7-3-1 Hongo, Bunkyo-ku, Tokyo, Japan; ^4^Department of Advanced Nursing Technology, Graduate School of Medicine, The University of Tokyo, 7-3-1 Hongo, Bunkyo-ku, Tokyo, Japan; ^5^Department of Nursing Administration, Graduate School of Medicine, The University of Tokyo, 7-3-1 Hongo, Bunkyo-ku, Tokyo, Japan; ^6^Department of Nursing, The University of Tokyo Hospital, 7-3-1 Hongo, Bunkyo-ku, Tokyo, Japan; ^7^Department of Diabetes and Metabolic Diseases, Graduate School of Medicine, The University of Tokyo, 7-3-1 Hongo, Bunkyo-ku, Tokyo, Japan

## Abstract

*Aim*. Callus is a risk factor, leading to severe diabetic foot ulcer; thus, prevention of callus formation is important. However, normal stress (pressure) and shear stress associated with callus have not been clarified. Additionally, as new valuables, a shear stress-normal stress (pressure) ratio (SPR) was examined. The purpose was to clarify the external force associated with callus formation in patients with diabetic neuropathy.* Methods*. The external force of the 1st, 2nd, and 5th metatarsal head (MTH) as callus predilection regions was measured. The SPR was calculated by dividing shear stress by normal stress (pressure), concretely, peak values (SPR-p) and time integral values (SPR-i). The optimal cut-off point was determined.* Results*. Callus formation region of the 1st and 2nd MTH had high SPR-i rather than noncallus formation region. The cut-off value of the 1st MTH was 0.60 and the 2nd MTH was 0.50. For the 5th MTH, variables pertaining to the external forces could not be determined to be indicators of callus formation because of low accuracy.* Conclusions*. The callus formation cut-off values of the 1st and 2nd MTH were clarified. In the future, it will be necessary to confirm the effect of using appropriate footwear and gait training on lowering SPR-i.

## 1. Introduction

Diabetic neuropathy is the most common complication of diabetes; approximately half of patients with diabetes have symptoms of diabetic neuropathy [1]. Loss of sensation is particularly important because it can allow the injury to go unnoticed, leading to foot ulcers. Diabetic foot ulcer is defined as cutaneous erosions characterized by a loss of epithelium that extends into or through the dermis to deeper tissues [2]; lifetime prevalence of diabetic foot ulcer is 15%–25% in the population with diabetes [3]. These foot ulcers seriously affect quality of life (QOL), reducing physical activity and increasing psychological stress [4]. Furthermore, a 2004 study estimated that diabetic ulcer-related costs averaged over $13,000 per episode, not including costs associated with psychosocial issues, decrease in QOL, and lost productivity [5]. In addition, even when an ulcer is successfully healed, the risk of recurrence is high, with reported rates ranging between 30% and 40% within one year [6, 7]. Therefore, prevention of foot ulcers is of paramount importance and has long been recognized as a priority by the International Working Group on the Diabetic Foot [8].

The pathogenesis of foot ulceration is a complex process in which many factors are involved. The most important factor appears to be peripheral neuropathy with a loss of sensation. However, neuropathy alone may not cause plantar ulceration [9]. Other risk factors are associated with developing diabetic foot ulcers, one of which is said to be the external force on the plantar [8]. Repeated normal stress (pressure) and shear stress during walking contribute to callus formation in the plantar region [10, 11]. Callus formation may lead to the development of foot ulcers and involves hyperkeratosis caused by excessive mechanical loading [2, 8, 12, 13]. The callus formation precedes ulcer formation in over 82% of patients with diabetic foot ulcers [14]. The relative risk for ulceration in a callused area was 11.0 compared with that of an area without callus [15, 16]. Once the foot ulcer occurs, its treatment will be difficult and take a long time. Therefore, prevention of the callus formation is important.

Patients with calluses are known to have significantly higher peak normal stress (pressure) during walking than patients without callus [17, 18]. Assuming that an average person walks approximately 10,000 steps a day, a callus may cause 18,600 kg of excess plantar normal stress (pressure) per day [19], highlighting the deleterious impact of calluses.

On the other hand, studies on shear stress are limited because the measurement of shear stress is technically difficult. Shear sensor technology has been still far from miniaturization to the point where it could accurately map shear load distribution. The shear stress has been commonly measured as ground reaction forces typically along, with a force platform providing resultant force acting on the outsole or barefoot because of technical difficulties. This fact appears to have acted as an almost complete barrier to practical, useful research relating to friction loads [20]. However, it is important to measure the in-shoe shear stress for considering the callus related factors. The shear stress of the callus formation area is hardly ever measured in patients with diabetes, and therefore, there are few studies on shear stress in patients with diabetes. As one of the few studies, patients with diabetes had higher shear stress under the first/second metatarsal head and lower shear stress under the third/fourth metatarsal head compared with healthy subjects [21, 22]. However, these studies used special type of shoes for measurement that could accommodate the thick sensor. It has been revealed that the shear stress of each metatarsal head differs in terms of direction and magnitude, depending on the difference of heel height using special type of shoes for measurement [23]. Therefore, it is important to perform measurements on the patient, while the patient is wearing his/her own ordinary footwear to identify causes associated with callus formation.

In the clinical setting, many patients redevelop calluses, despite wearing tailor-made footwear developed with a view for preventing callus. Tailor-made footwear that is declared for the callus prevention is available, the effectiveness of which has already been demonstrated [24, 25]; however, Scirè et al. also showed that the callus recurred in 41% patients, even if they wore therapeutic footwear [26]. The callus recurred in 24 of 31 patients, even if they received foot care and wore recommended footwear, according to the diabetic foot care program [27]. From these results, it is considered that the causes of callus have not been completely excluded. It is assumed that the callus has been intervened without considering shear stress during walking. Although repeated normal stress (pressure) and shear stress during walking contribute to callus formation, many studies on intervention were focusing only on normal stress (pressure). Shear stress is another essential factor to consider for developing more effective care.

It is also unclear how much the external force on plantar should be decreased, despite many studies being available on normal stress (pressure) analysis. Identification of a cut-off value of the normal stress (pressure) has been attempted for foot ulcer prevention, but the cut-off value has less accuracy [16, 28]. Moreover, a study of a cut-off value of the shear stress is hardly available. The cut-off value of the normal stress (pressure) and shear stress on callus formation prevention has not been studied. As for the future clinical image, in the next procedure of intervention, the external forces will be adjusted referring to the cut-off value. If the callus recurs, the external forces will be readjusted to the smaller value of the current value.

The purpose of this study was to clarify external forces associated with callus formation in patients with diabetic neuropathy and to identify the cut-off value of the forces. First, differences in normal stress (pressure) and shear stress in callus formation region and noncallus formation region will be clarified in patients with diabetic neuropathy. Second, the external force cut-off value for callus formation will be identified in patients with diabetic neuropathy.

## 2. Research Design and Methods

### 2.1. Research Design

The walking measurement was cross-sectional study, and observation of callus formation was carried out as longitudinal study.

### 2.2. Participants

This survey was conducted at the Diabetic Foot Outpatient Clinic at the University of Tokyo Hospital from April to October 2015. Sixty-four patients with diabetes who visited this outpatient clinic were recruited. Inclusion criteria were patients with diabetic neuropathy ≥20 years old who could walk without aid. Participants who had a current diabetic foot ulcer and a history of more proximal lower limb of metatarsophalangeal (MTP) joint amputation were excluded. The survey protocol was approved by the Ethical Committee of the Graduate School of Medicine, The University of Tokyo (#10797).

**Figure 1:**
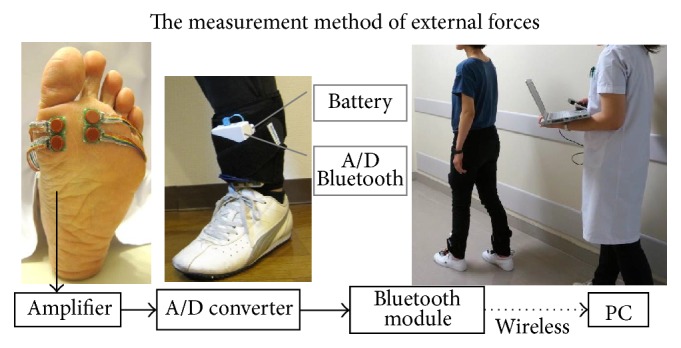
The state of attaching the sensors at the 1st and 2nd metatarsal head.

Aim of this outpatient clinic is to prevent diabetic foot ulcers, and therefore, most patients experience symptoms of the foot or are interested in the prevention of complications. After general examination in the diabetic foot outpatient clinic including regular callus removal, written informed consent was obtained from the participant. Sensors were attached and walking measurement was executed. The patient walked approximately 50 m as a practice to confirm that there was no pain and no interfering in walking by the sensor attachment. The patient then walked about 15 m twice as the measurement walk, and the researcher recorded all sensor data ensuring that sensors were operating during the measurement. An assistant measured 15 m walking time using a stopwatch and walked diagonally behind the patient to prevent them from falling or other adverse events (Figure 1).

The participants were asked to wear “usual shoes” when they visited the Diabetic Foot Outpatient Clinic. It was assumed that those shoes were worn for the longest time, such as during exercise therapy. All participants were provided with standardized socks to use during the measurement. When the pretest participants had come with wearing tight or long socks, attached sensors had been often displaced in the pretest. In these situations, remeasurement and confirmation of the position of attaching sensor had been repeated. Thus, the patients' own socks could not be used to shorten the investigation time and to reduce the burden of the patients.

The presence or absence of callus was checked after one month again, which means the formation of callus check; this is also the definition of callus formation region in this study.

### 2.3. Data Collection

#### 2.3.1. Callus

The definition of callus is not clear universally; therefore the presence/absence of callus is determined in the clinical setting using expert opinion in this study. That is, in this study, a callus was defined by plate-shaped hyperkeratosis observed by two expert nurses in foot care. All calluses were removed in the diabetic foot clinic using a corn cutter, and walking was measured afterward.

The presence or absence of callus formation was checked again after one month in all patients, because, generally, a callus would have recurred within one month. The region of such callus was classified into the callus formation group if callus recurred. If the callus does not recur in the region, it will be excluded from the analysis. The region of noncallus was classified into the noncallus formation group if a callus did not develop after one month. If a callus developed in the noncallus region during the follow-up for 1 month, it was also excluded from the analysis, since it could not be determined that the measured gait and foot condition were related to callus formation.

#### 2.3.2. External Forces

**Figure 2:**
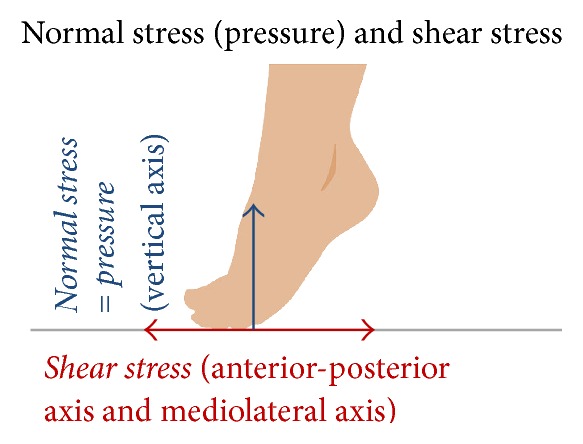
Normal stress (pressure) is vertical axis. Shear stress is the resultant force of anterior-posterior axis and mediolateral axis.

In this study, in-shoe normal stress (pressure) and shear stress of the 1st, 2nd, and 5th MTH were measured; these are the predilection regions of callus and foot ulcers. Reliability and validity of the system for measuring in-shoe plantar normal stress (pressure) and shear stress have already been verified [29]. These in-shoe normal stress (pressure) and two axes' shear stress were measured by ShokacChip™, which was newly developed by Touchence Inc. and released in 2013. ShokacChip is a tactile sensor with small-high sensitivity based on MEMS (Micro Electro Mechanical Systems) technology. High sensitivity is realized for three-dimensional axes by processing three piezoelectric elements and locating them at three-dimensional axes on the 2 mm^2^ chip. Sensor size is *φ*10.0 mm × 1.3 mm (t); it is very small and thin [30]. Thus, it can measure the in-shoe external forces of each MTH. Calibration using a load-compensating device has already been done before shipment of the sensors. It has been guaranteed that this sensor is not necessary to calibrate in each time of measurement on the characteristic of the sensor. However, calibration was done by rising a foot to each foot before the measurement in a state of wearing shoes, because the sensor caught some stresses when patients wore shoes. Subject's plantar was fixed perpendicular to the ground and the sensor was attached to be parallel to the ground at the region of ranging from the MTH top to the base of toe by double sided tape. The fixed method of the sensors was considered before pretest. Additionally, location of the sensor was checked visually after each measurement. Shear stress is the resultant force of anterior-posterior axis and mediolateral axis on the assumption of some sensor shift (Figure 2). From results of pretests, measurements should use two sensors in each region. Only two regions could be measured per foot in one measurement, because four sensors, at a maximum, could be used per foot by the sensor circuit system hardware restriction. The callus regions were preferentially selected for measurement, and the noncallus regions were selected at random in these three regions. If a noncallus patient could be matched by sex and age (±3 years), the same regions were measured in the callus patient. If a foot had three calluses, measurements were conducted twice by changing the attachment of the sensor because the number of sensors was limited. The noncallus region will be excluded when one foot has callus region and noncallus region, because noncallus region might be also affected by callus. It is considered that the cause of callus formation of toe is the contact with shoes. These calluses are clearly different cause to callus of metatarsal heads. Therefore, feet with calluses of toe are not excluded.

Peak plantar normal stress (pressure) (PP), normal stress (pressure) time integral (PI), peak shear stress (PSS), and shear stress integral (SSI) of each gait cycle were calculated from the recorded force profile; these variables have been investigated in many previous studies of external force [31–33]. In addition, during measurements, we newly noticed that some callus patients had a high shear stress in spite of the not so high normal stress (pressure). One previous study [34] examined the combinations of normal stress (pressure) and shear stress; they concluded that skin breakdown occurred at higher shear load levels in animal experiment. However, it was indicated in the results that loading pad was slipped and skin breakdown did not occur in the combination of some shear stress and low normal stress (pressure) loading. From these results, we considered that the balance of normal stress (pressure) and shear stress is important. Thus, shear stress-normal stress (pressure) ratios (SPR) were calculated by dividing shear stress by normal stress (pressure), concretely, peak values (SPR-p) and time integral values (SPR-i).

#### 2.3.3. Patient Characteristics

Data regarding age, sex, height, weight, foot length, and width (standing position) and usual daily activities were obtained from medical records or by interview. Hallux valgus, bunionette, claw toe, and hammer toe were regarded as foot deformities and identified by the visual inspection of well-trained nurse.

Patients were diagnosed with diabetic neuropathy if two of the following three items were fulfilled: (1) sensory symptoms considered to be due to diabetic neuropathy, (2) bilaterally decreased or absent ankle reflex, and (3) decreased vibratory sensation in bilateral medial malleoli [35]. The sensory symptoms considered to be due to diabetic neuropathy were clarified during the interview, whereas the bilaterally decreased or absent ankle reflex was examined with the patient in a kneeling position. Decreased vibratory sensation in bilateral medial malleoli was examined using an AC128 tuning fork for evaluation of vibration sense. The total time span in which the patient felt vibratory sensation was evaluated, and a duration of <10 s was considered as decreased sensation [35]. In addition, the results of the monofilament test were confirmed using medical records; this test was performed on the basis of the Practical Guidelines of International Working Group on the Diabetic Foot using 5.07 Semmes-Weinstein monofilament (ARKRAY Inc., Tokyo, Japan). Data regarding duration of diabetes, hemoglobin A1c (HbA1c), foot ulcer history, and foot amputation history were obtained from medical records or by interview. Angiopathy data were collected from medical records, and an ankle-brachial index (ABI) <0.9 was regarded as angiopathy [36].

### 2.4. Data Analysis

The variables of the external forces were obtained by averaging a total 15 steps after removing the initial three steps and the final three steps. Data processing was performed using MATLAB R2012a (The Math Works, Inc., MA, USA).

Descriptive data were expressed as means ± standard deviations for continuous variables and *n* (%) for categorical variables. Statistical analyses were performed using IBM SPSS Statistics ver. 23.0 (Chicago, IL, USA). Statistical significance was set at *p* = 0.05. Patient characteristics were compared between the callus formation group and noncallus formation group using Student's *t*-test and Fisher's exact test. For comparison of the external forces in callus formation regions and noncallus formation regions, Student's *t*-test was used.

Receiver operating characteristic (ROC) curves were drawn for all external force variables, and the area under curve (AUC) was calculated. The optimal cut-off point was determined with the specificity being approximately 0.8. Positive predictive values (PPVs) and negative predictive values (NPVs) were also calculated. The reason why the specificity has priority is that the callus can be removed unlike a foot ulcer even if it occurs once. On the other hand, intervention to improve the external force (e.g., the introduction of custom-made footwear) has a large burden on medical staff and patients. Therefore, the cut-off value was examined to avoid excessive intervention.

## 3. Results

Sixty-four patients with diabetes were attended to at the Diabetic Foot Outpatient Clinic during the observation period, and fifty-nine patients with diabetic neuropathy participated in the survey. Five patients were excluded because the patient (1) did not have neuropathy (*n* = 1), (2) used a wheelchair (*n* = 1), (3) had a current diabetic foot ulcer (*n* = 1), (4) had a history of bilateral knee amputation (*n* = 1), and (5) was unable to provide consent for participation (*n* = 1).

Twenty patients (33.9%) had more than one callus in the target region, whereas the number of patients without callus was 39 (66.1%). Measurement regions were 244 regions; four patients had 3 calluses in one foot. Thirty-eight regions (15.6%) were excluded from the analysis because other regions had a callus in one foot. As for the 1st MTH, the number of callus formation feet was 13 (23.6%), whereas the number of noncallus formation feet was 42 (76.4%). As for the 2nd MTH, the number of callus formation feet was 16 (22.2%), whereas the number of noncallus formation feet was 56 (77.8%). As for the 5th MTH, the number of callus formation feet was 21 (26.6%), and the number of noncallus formation feet was 58 (73.4%).

All calluses had recurred by the time of the next follow-up (1 month later), whereas no calluses were seen in any of the noncallus regions at the follow-up. Therefore, all regions of callus removal were assigned to regions of “callus formation,” and all regions with no calluses were assigned to regions of “noncallus formation” in this study.

Measurement survey took about 15 min; approximately five minutes was required for follow-up survey. No adverse events were observed during the survey.

The patient characteristics for each MTH are shown in Table 1.

In the 1st MTH results, the foot deformity was significantly higher in the callus formation group. In the 2nd MTH results, there were significantly more female patients, light weight patients, patients with low HbA1c, and patients with foot deformity in the callus formation group. In the 5th MTH results, the two groups had no significant difference.

**Figure 3:**
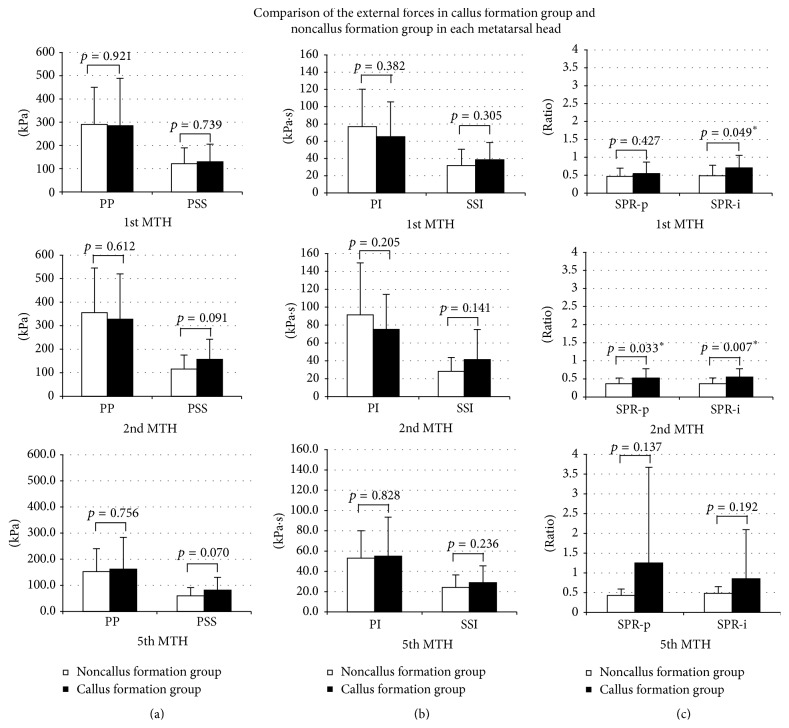
MTH: metatarsal head. (a) Peak normal stress (pressure) (PP) and peak shear stress (PSS), (b) normal stress (pressure) time integral (PI) and shear stress time integral (SSI), and (c) shear stress/normal stress (pressure) ratio of peak value (SPR-p) and shear stress/normal stress (pressure) ratio of time integral value (SPR-i).

The results for the 1st MTH are shown in Figure 3. Single variables of normal stress (pressure) and shear stress did not differ significantly; only SPR-i was significantly higher in the callus formation group. The results for the 2nd MTH are shown in 2nd row of Figure 3. Single variables of normal stress (pressure) and shear stress did not differ significantly; only SPR-p and SPR-i were significantly higher in the callus formation group. The results for the 5th MTH are shown in 3rd row of Figure 3. No variables differed significantly; however, PSS had a tendency toward being high in the callus formation group (*p* = 0.070).

**Figure 4:**
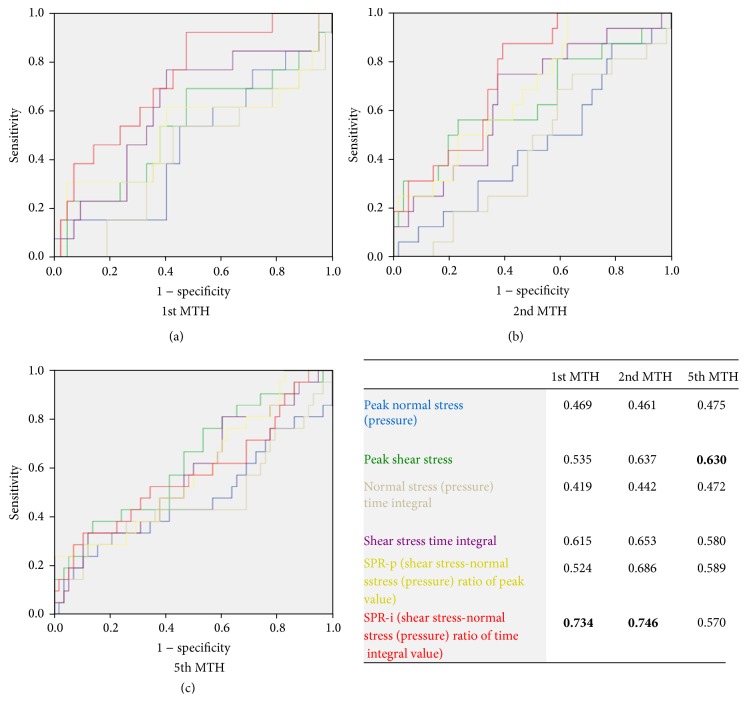
Receiver operating characteristics (ROC) curve of all external force variables in each metatarsal head. (a) 1st MTH, (b) 2nd MTH, and (c) 5th MTH.

ROC curves for the 1st MTH are shown in Figure 4(a). The cut-off value of SPR-i was 0.60 (sensitivity, 0.54; specificity, 0.76) for the 1st MTH. The ROC curves for the 2nd MTH are shown in Figure 4(b). The cut-off value of SPR-i was 0.50 (sensitivity, 0.44; specificity, 0.80) in the 2nd MTH. ROC curves for the 5th MTH are shown in Figure 4(c). For the 5th MTH, variables pertaining to the external forces could not be determined to be indicators of callus formation, because the AUC of all variables was <0.7, although PSS in AUC was 0.63.

## 4. Conclusions

This is the first study that measures the in-shoe external force of the 1st, 2nd, and 5th metatarsal heads of the foot. Since this measurement had been enabled, it was clarified that callus formation of the 1st and 2nd metatarsal head were related to high shear stress time integral/normal stress (pressure) time integral (SPR-i) rather than the single variables of normal stress (pressure) and shear stress. Additionally, the cut-off values for each region were found to be different.

The measurement of the actual in-shoe normal stress (pressure) and shear stress on the plantar has been associated with technical difficulties. In this study, the in-shoe external forces were measured in the clinic setting, since it became possible to conduct measurements in any shoe utilizing the thin and small sensor that was recently developed. In addition, all patients with diabetic neuropathy could participate in this survey without adverse events. Furthermore, the in-sole type sensor systems used in the previous studies were unclear whether the systems measured external force applied to the callus region since it had been difficult to confirm the sensor attached to the callus region [23, 37]. In this study, this problem was solved by directly attaching the sensor on the target area on the plantar. Hence, this study is highly important because normal stress (pressure) and shear stress of the callus regions could be accurately measured simultaneously.

It was revealed that SPR-i is the new indicator of callus formation; that is, dividing shear stress with normal stress (pressure) indicated the balance between shear stress and normal stress (pressure). The balance of the external forces had not been previously investigated. With higher shear stress-normal stress (pressure) ratio, as the high shear stress applies with a certain degree of normal stress (pressure), mechanical stress on the skin will become increased, and the body attempts to protect irritated skin by forming a hyperkeratotic lesion, such as a corn or a callus [11]. With lower shear stress-normal stress (pressure) ratio, when high normal stress (pressure) applies with small shear stress, it is considered that the callus, which is the normal physiologic response of the skin, will be hardly formed, since the external forces are applied to the subcutaneous tissue rather than being exerted on the skin surface. Calluses form as a result of hyperproliferation and incomplete differentiation of epidermal keratinocytes and increased expression of adhesion molecules in the epidermis [38]. Forces and time integration were associated with the callus formation rather than the maximum forces, because the time integral ratio had a high accuracy than the peak ratio.

Two reasons could be considered as SPR-i of the 5th metatarsal head did not have significant difference between callus formation group and noncallus formation group. First, sample size of the 5th metatarsal head was limited. If enough patients with the 5th metatarsal head callus will participate, SPR of the 5th metatarsal head might also become the indicator. More patients were needed for the 5th metatarsal head, because external force of the 5th metatarsal head had large variability and small value of difference compared with other regions. Second, peak shear stress affected the callus formation of 5th metatarsal head rather than SPR-i. Mechanical stress might not be absorbed and off-loaded in the subcutaneous tissue, because the 5th metatarsal head has thin subcutaneous tissue comparing other two regions. Therefore, peak shear stress might affect the callus formation of 5th metatarsal head rather than the balance of normal stress (pressure) and shear stress (SPR-i).

According to these results, there was no significant difference in the normal stress (pressure) and shear stress variables between the callus formation group and noncallus formation group in all regions. It was only observed that peak shear stress tended toward being high in the 2nd and 5th metatarsal head. In some previous studies, where the calluses were measured without prior removal, the normal stress (pressure) and shear stress were significantly higher in the callus group. Some studies had shown that the normal stress (pressure) decreased after removing the callus [19, 39]. Therefore, it is natural that single variables of normal stress (pressure) and shear stress had no significant differences in the absence of hyperkeratosis.

The cut-off values of external force on plantar associated with callus formation were found; SPR-i of the 1st metatarsal head was 0.60 and SPR-i of the 2nd metatarsal head was 0.50. The cut-off value of SPR-i for the 2nd metatarsal head was lower than that for the 1st metatarsal head. This may be the reason why the 2nd metatarsal head was associated with a higher normal stress (pressure) than that associated with the 1st metatarsal head. In general, the center of normal stress (pressure) is applied to the 2nd metatarsal head during walking, and the center of normal stress (pressure) during the push-off phase, which is higher, often applies to this region in patients with diabetes [40]. Thus, the normal stress (pressure) in the 2nd metatarsal head was higher than the normal stress (pressure) of the 1st metatarsal head.

In the 5th metatarsal head, individual differences were more pronounced than for the other regions. The reason might be that the moving of the center of gravity from the heel to the 5th metatarsal head during walking hardly connects to the propulsion. Therefore, the external force could not be determined to be the sole indicator of callus formation in the 5th metatarsal head.

In various previous studies, normal stress (pressure) had been associated with foot ulcer development [16, 38, 41], and therefore, the following process was considered. Calluses were developed under the influence of high SPR-i. High normal stress (pressure) was applied to the callus region by hyperkeratosis [19], and foot ulcer subsequently developed causing subcutaneous tissue damage due to high normal stress (pressure).

The patients of this survey were limited to mild neuropathy and mild foot deformity patients. Only one patient had a history of amputation. Therefore, the results of this study might not be applicable to patients with a progressed neuropathy, severe foot deformity, and amputation history. It will be necessary to confirm it in the future whether this result is applicable to such a high risk subject.

It is limitation that the walking measurements were taken on one occasion only. It was necessary to check that the gait features were not changed, when observing callus formation after one month. However, it was difficult because this study was carried out during medical practice. This study was conducted on the assumption that the gait features were not changed during this one month.

For the first time, each region of the normal stress (pressure) and shear stress wearing the patient's own shoes was measured in a clinical setting. As a result, the following two points were revealed. First, callus formation in the 1st and 2nd metatarsal head is related to high shear stress time integral/normal stress (pressure) time integral (SPR-i) rather than the single variables of normal stress (pressure) and shear stress. Peak shear stress had a tendency towards being high in the callus formation group in the 5th metatarsal head. Second, the external force cut-off values were found to differ in each site, SPR-i of the 1st metatarsal head being 0.60 and SPR-i of the 2nd metatarsal head being 0.50. External force could not be determined to be a sole indicator of callus formation in the 5th metatarsal head. Considering the results presented here, intervention based on cut-off values of SPR-i of each metatarsal head will be effective. In future, it will be necessary to confirm the effect of using appropriate footwear and gait training on lowering external forces associated with callus formation.

## Figures and Tables

**Table 1:** Patient characteristics for each metatarsal head observation.

	1st metatarsal head			2nd metatarsal head			5th metatarsal head		
	Noncallus formation group	Callus formation group	*p* value	Noncallus formation group	Callus formation group	*p* value	Noncallus formation group	Callus formation group	*p* value
Number of feet	42	13		56	16		58	21	
Patient characteristics									
Age (y)	66.0 ± 11.2	63.7 ± 8.6	0.450^1^	66.7 ± 10.8	65.1 ± 7.8	0.517^1^	69.3 ± 10.2	67.0 ± 13.4	0.471^1^
Sex			0.053^2^			0.004^2*∗*^			0.599^2^
Male	20 (47.6)	2 (15.4)		30 (53.6)	2 (12.5)		38 (65.5)	12 (57.1)	
Female	22 (52.4)	11 (84.6)		26 (46.4)	14 (87.5)		20 (34.5)	9 (42.9)	
Height (m)	1.61 ± 0.1	1.56 ± 0.1	0.133^1^	1.61 ± 0.09	1.57 ± 0.13	0.289^1^	1.62 ± 0.09	1.64 ± 0.13	0.504^1^
Weight (kg)	66.8 ± 17.5	66.9 ± 23.9	0.999^1^	68.0 ± 17.7	58.3 ± 9.3	0.005^1*∗*^	63.1 ± 14.6	65.1 ± 14.8	0.595^1^
BMI	25.5 ± 5.4	26.8 ± 6.2	0.495^1^	25.9 ± 5.2	23.7 ± 3.9	0.068^1^	24.0 ± 4.4	24.2 ± 4.0	0.856^1^
HbA1c: NGSP (%)	6.9 ± 1.3	6.7 ± 0.7	0.134^1^	7.1 ± 1.0	6.3 ± 0.5	<0.001^1*∗*^	6.7 ± 0.8	6.9 ± 1.2	0.507^1^
HbA1c: IFCC	52 ± 10	50 ± 5		54 ± 8	45 ± 4		50 ± 6	52 ± 9	
(mmol/mol)									
Diabetes duration (y)	15.6 ± 10.3	13.5 ± 7.5	0.683^1^	17.9 ± 9.9	13.4 ± 10.7	0.143^1^	15.7 ± 11.3	15.8 ± 7.1	0.986^1^
Monofilament test	5 (11.9)	1 (7.7)	1.000^2^	4 (7.1)	0 (0.0)	1.000^2^	5 (8.6)	4 (19.0)	0.236^2^
(abnormal)									
History of diabetic	2 (4.8)	0 (0.0)	1.000^2^	0 (0.0)	0 (0.0)		2 (3.4)	0 (0.0)	1.000^2^
Foot ulcer									
Deformity	9 (21.4)	10 (76.9)	<0.001^2*∗*^	7 (12.5)	7 (43.8)	0.010^2*∗*^	8 (13.8)	5 (23.8)	0.314^2^
Angiopathy	2 (4.8)	1 (7.7)	0.562^2^	2 (3.6)	1 (6.3)	0.535^2^	4 (6.9)	1 (4.8)	1.000^2^

Mean ± SD, *n* (%) ^*∗*^ *p* < 0.05  ^1^ *t*-test, ^2^Fisher's exact test.
